# Requirements for human haematopoietic stem/progenitor cells

**DOI:** 10.1111/cpr.13152

**Published:** 2021-12-22

**Authors:** Xue Nan, Bowen Zhang, Jie Hao, Wen Yue, Boqiang Fu, Mingyi Qu, Yu Zhang, Haiyang Wang, Fang Fang, Jun Wei, Qiyuan Li, Shijun Hu, Junying Yu, Yingdai Gao, Qifa Liu, Jiani Cao, Lei Wang, Yaojin Peng, Huanxin Zhu, Lingmin Liang, Aijin Ma, Jiaxi Zhou, Tongbiao Zhao, Xuetao Pei

**Affiliations:** ^1^ South China Research Center for Stem Cell & Regenerative Medicine SCIB Guangzhou China; ^2^ South China Cell & Stem Cell Bank SCIB Guangzhou China; ^3^ Chinese Society for Stem Cell Research Shanghai China; ^4^ National Stem Cell Resource Center State Key Laboratory of Stem Cell and Reproductive Biology Institute of Zoology Institute for Stem Cell and Regeneration Chinese Academy of Sciences Beijing China; ^5^ University of Chinese Academy of Sciences Beijing China; ^6^ Beijing Institute for Stem Cell and Regenerative Medicine Beijing China; ^7^ National Institute of Metrology Beijing China; ^8^ Department of State‐owned Assents and Laboratory Management Capital Medical University Beijing China; ^9^ Zephyrm Biotechnologies Co Ltd, Beijing China; ^10^ China National GeneBank Shenzhen China; ^11^ Department of Cardiovascular Surgery of the First Affiliated Hospital & Institute for Cardiovascular Science Collaborative Innovation Center of Hematology State Key Laboratory of Radiation Medicine and Protection Medical College Soochow University Suzhou China; ^12^ Nuwacell Biotechnology Co., Ltd Hefei China; ^13^ Institute of Hematology and Blood Diseases Hospital Chinese Academy of Medical Sciences & Peking Union Medical College Tianjin China; ^14^ Nanfang Hospital Southern Medical University Guangzhou China; ^15^ Beijing Technology and Business University Beijing China

**Keywords:** applications, human haematopoietic stem/progenitor cells, requirements, standard

## Abstract

‘Requirements for human haematopoietic stem/progenitor cells’ is the first set of guidelines on human haematopoietic stem/progenitor cells in China, jointly drafted and agreed upon by experts from the Chinese Society for Stem Cell Research. This standard specifies the technical requirements, inspection methods, inspection rules, instructions for usage, labelling requirements, packaging requirements, storage requirements and transportation requirements for human haematopoietic stem/progenitor cells, which is applicable to the quality control for human haematopoietic stem/progenitor cells. We hope that publication of these guidelines will promote institutional establishment, acceptance and execution of proper protocols, and accelerate the international standardization of human haematopoietic stem/progenitor cells for applications.

## 1 SCOPE

This document specifies the technical requirements, test methods, test regulations, instructions for use, labelling requirements, packaging requirements, storage requirements, transportation requirements and waste disposal requirements of human haematopoietic stem/progenitor cells.

This document is applicable for the production and testing of human haematopoietic stem/progenitor cells.

This document does not apply to haematopoietic stem cells involved in the *Technical management specifications for haematopoietic stem cell transplantation* and *Cord blood haematopoietic stem cell bank management specifications (Trial)*.

## 2 NORMATIVE REFERENCES

The following content constitutes indispensable articles of this standard through normative reference. For dated references, only the edition cited applies. For undated references, only the latest edition (including all amendments) applies.
*GB/T 6682 Water for analytical laboratory use—Specification and test methods*
*WS 213 Diagnosis for hepatitis C*
*WS 273 Diagnosis for syphilis*
*WS 293 Diagnosis for HIV/AIDS*
*T/CSCB 0001 General requirements for stem cells*
*Pharmacopoeia of the People's Republic of China*
*National Guide to Clinical Laboratory Procedures*

## 3 TERMS AND DEFINITIONS

For the purpose of this document, the terms and definitions in T/CSCB 0001, T/CSCB 0002 and following terms and definitions apply to this document.

### 3.1 Human haematopoietic stem cells

Stem cells that have the ability to self‐renew and differentiate into all mature blood cell types.

### 3.2 Human haematopoietic progenitor cells

Progenitor cells that have the ability to differentiate into multiple or a particular lineage of blood cells.

### 3.3 Haematopoietic colony

Cell clusters or colonies containing recognizable progeny generated from individual haematopoietic progenitor cells cultured in a semi‐solid medium containing the appropriate cytokines.

## 4 ABBREVIATIONS

BFU‐E: burst‐forming unit‐erythroid

CD: cluster of differentiation

CFU‐GEMM: colony‐forming unit‐granulocyte‐erythroid‐macrophage‐megakaryocyte

CFU‐GM: colony‐forming unit‐granulocyte‐macrophage

EBV: Epstein‐Barr virus

HBV: hepatitis B virus

HCMV: human cytomegalovirus

HCV: hepatitis C virus

HIV: human immunodeficiency virus

HTLV: human T‐cell lymphotropic virus

NOD‐SCID: non‐obese diabetic‐severe combined immunodeficient

STR: short tandem repeat

TP: treponema pallidum

## 5 TECHNICAL REQUIREMENTS

### 5.1 Source materials and ancillary materials

5.1.1 The requirements of T/CSCB 0001 shall be followed.
5.1.2 To ensure the safety of the donor and the donated cells, the process for donor evaluation and screening, cell collection, transportation and receipt shall be standardized.
5.1.3 The donor shall be screened for HIV, HBV, HCV, HTLV, EBV, HCMV and TP, and the results shall be documented.

### 5.2 Primary quality attributes

#### 5.2.1 Cell morphology

Cells grown under suspended conditions shall be round and uniform in size. Cells shall be round, and exhibit high nuclear‐to‐cytoplasmic ratio, light blue cytoplasm and no granular structure in the cytoplasm after the Wright‐Giemsa staining.

#### 5.2.2 Chromosome karyotype

The normal karyotype shall be 46, XY or 46, XX.

#### 5.2.3 Cell viability

Shall be ≥85% before cryopreservation and ≥70% after resuscitation.

#### 5.2.4 Cell markers

The expression of CD34 shall be ≥80% of the cell population.

#### 5.2.5 Colony‐forming unit assays

Number of total colonies shall be ≥10 per 10^3^ cells, and number of CFU‐GEMM colonies shall be ≥1 per 10^3^ cells.

#### 5.2.6 Haematopoietic reconstitution assays

Sixteen weeks post‐transplantation into NOD‐SCID Il2rg^null^ mice, human CD45^+^ cell reconstitution levels shall be ≥5%, and human CD45^+^CD19^+^ cells, CD45^+^CD3^+^ cells, CD45^+^CD33^+^ cells and CD45^−^CD235a^+^ cells shall be positive in total peripheral blood mononuclear cells of the recipient mice.

#### 5.2.7 Microorganisms

Fungi, bacteria, mycoplasma, HIV, HBV, HCV, HTLV, EBV, HCMV and TP shall be negative.

### 5.3 Process control

5.3.1 The process of cell expansion, cryopreservation and resuscitation shall follow the requirements of T/CSCB 0001.
5.3.2 The identity of the cells shall match with that of donor cells by STR analysis.

## 6 TEST METHODS

### 6.1 Cell morphology

Observe the morphology of cells using a microscope. Cell staining shall be performed following the *National Guide to Clinical Laboratory Procedures*.

### 6.2 Chromosome karyotype

The method in the *Pharmacopoeia of the People's Republic of China* shall be followed.

### 6.3 Cell viability

The method in Appendix A shall be followed.

### 6.4 Cell markers

The method in Appendix B shall be followed.

### 6.5 Colony‐forming unit assays

The method in Appendix C shall be followed.

### 6.6 Haematopoietic reconstitution assays

The method in Appendix D shall be followed.

### 6.7 Microorganisms

#### 6.7.1 Fungi

The method ‘1101 sterility test’ in the *Pharmacopoeia of the People's Republic of China* shall be followed.

#### 6.7.2 Bacteria

The method ‘1101 sterility test’ in the *Pharmacopoeia of the People's Republic of China* shall be followed.

#### 6.7.3 Mycoplasma

The method ‘3301 sterility test’ in the *Pharmacopoeia of the People's Republic of China* shall be followed.

#### 6.7.4 HIV

The nucleic acid test method in WS 293 shall be followed.

#### 6.7.5 HBV

The nucleic acid test method in the *National Guide to Clinical Laboratory Procedures* shall be followed.

#### 6.7.6 HCV

The nucleic acid test method in WS 213 shall be followed.

#### 6.7.7 HTLV

The nucleic acid test method in the *National Guide to Clinical Laboratory Procedures* shall be followed.

#### 6.7.8 EBV

The nucleic acid test method in the *National Guide to Clinical Laboratory Procedures* shall be followed.

#### 6.7.9 HCMV

The nucleic acid test method in the *National Guide to Clinical Laboratory Procedures* shall be followed.

#### 6.7.10 TP

The nucleic acid test method in WS 273 shall be followed.

## 7 INSPECTION RULES

### 7.1 Sampling method

7.1.1 Cells produced from the same production cycle, same production line, same source, same passage and same method are considered to be the same batch.
7.1.2 Three smallest units of packaging shall be randomly sampled from the same batch.

### 7.2 Quality inspection and release

Each batch of cell preparation shall be subject to the quality inspection before release. The quality inspection items shall include all the attributes specified in 5.2. The inspection reports shall be attached.

### 7.3 Review inspection

Review inspection shall be performed by professional cytological testing institutions/laboratories as necessary.

### 7.4 Decision rules

Products that pass all requirements in 5.2 for the quality inspection and quality review inspection are considered to be qualified. Products that do not meet these criteria should be considered unqualified.

## 8 INSTRUCTION FOR USAGE

The instructions for usage shall include, but not limited to:

- Product name;
- Passage number;
- Cell numbers;
- Production date;
- Lot number;
- Production organization;
- Storage conditions;
- Shipping conditions;
- Operation manual;
- Execution standard number;
- Manufacturing address;
- Contact information;
- Postal code;
- Matters that need attention.

Note: Upon user’s requirement, endotoxin test results can be provided.

## 9 LABELS

The label shall include, but not limited to:

- Product name;
- Passage number;
- Cell number;
- Lot number;
- Production organization;
- Production date.

## 10 PACKAGE, STORAGE AND TRANSPORTATION

### 10.1 Package

The appropriate materials and containers shall be selected to ensure maintenance of the primary quality attributes of human haematopoietic stem/progenitor cells.

### 10.2 Storage

10.2.1 T/CSCB 0001 shall be followed.
10.2.2 Productions should be stored at a temperature below ‐130°C.

### 10.3 Transportation

10.3.1 T/CSCB 0001 shall be followed.
10.3.2 Cryopreserved cell products shall be transported in dry ice or at temperature below ‐130°C. Non‐cryopreserved cell products shall be transported at temperature between 2 and 8°C.

## 11 WASTE DISPOSALS

Waste that arises during human haematopoietic stem/progenitor cell production and detection shall be disposed according to the regulations in T/CSCB 0001.

## APPENDIX A

### (Normative)

Cell Viability Test and Cell Counting

#### A.1 Instruments

A.1.1 Microscope

A.1.2 Haemocytometer (XB‐K‐25)

#### A.2 Reagents

Unless otherwise specified, all reagents shall be of analytical purity. The water used in all tests shall be deionized.

A.2.1 Phosphate‐buffered saline: pH 7.4.

A.2.2 Trypan blue solution: concentrated stock solution is diluted to 0.4% (W/V) with phosphate‐buffered saline (A.2.1) for use.

#### A.3 Procedure

A.3.1 Preparing single‐cell suspension

Collect the cells to be tested and resuspend the cells in phosphate‐buffered saline (A.2.1).

A.3.2 Trypan blue staining

Dilute the cell suspension (A.3.1) in trypan blue solution (A.2.2) at 1:1, and mix well.

A.3.3 Cell counting

Put coverslips on each chamber of a clean haemocytometer (A.1.2). Transfer 10 µL trypan blue/cell suspension (A.3.2) to the edge of the coverslip, allowing the cell suspension to fully fill the chambers under the coverslip without over‐ or underfill. Repeat with the second chamber. Let stand for 30 seconds, and count all cells (stained and unstained) and the stained cells in each chamber under microscope (A.1.1).

Repeat steps A.3.2 to A.3.3 once.

A.3.4 Calculating the viability

#### A.4 Calculation

The cell viability can be calculated with the following formula (A.1):

S = (M‐D)/M × 100% (A.1)

Note:

S—cell viability

M—total cell number

D—stained cell number

Calculate the average viability of two repeats. This result is the average cell viability.

#### A.5 Precision

Under the same condition, the absolute deviation of the two repeats should not exceed 10% of the arithmetic mean.

## APPENDIX B

### (Normative)

Cell markers and flow cytometry analysis

### B.1 Instruments

B.1.1 Flow cytometer

B.1.2 Horizontal centrifuge

B.1.3 Electronic balance

### B.2 Reagents

All reagents in this method are of analytical purity. Unless otherwise specified, the water used in all tests is the level 1 water as specified in GB/T 6682.

B.2.1 Phosphate‐buffered saline (PBS): pH 7.4.

B.2.2 Bovine serum albumin (BSA): purity ≥98%

B.2.3 Anti‐human CD34 antibody and isotype control.

B.2.4 Prepare solutions needed for flow cytometry assay using electronic balance (B.1.3): washing buffer, fixation buffer, blocking/permeabilization buffer and antibody dilution buffer.

### B.3 Procedure

B.3.1 Sample preparation

Harvest single cells by centrifugation at 300 g for 5 min. Discard the supernatant. Wash the cell samples with an appropriate volume of wash solution, then collect samples by centrifuging at 300 g for 5 min, discard the supernatant and repeat 2 times.

B.3.2 Antibody incubation

Divide the cell suspension into two equal aliquots for isotype control and test. Dilute and stain the cells with anti‐human CD34 antibody (labelled as sample group) and isotype control (labelled as negative control group) separately according to their instructions/manuals. Wash the cells with washing buffer twice, collect samples by centrifuging at 300 g for 5 min, and discard the supernatant.

B.3.3 Flow cytometry analysis

Resuspend the cells with the washing solution, and then transfer the cell suspension to the tube through a 40‐μm filter, and test sample on the flow cytometer according to the application manual.

B.3.4 Gating strategies

First, exclude events of debris, dead cells and untargeted cell populations by drawing a gate (gate 1) according to estimated cell size (FSC) and granularity (SSC). Next, by comparing the test and isotype control, place the gate for positive staining population (gate 2) to exclude the cells not labelled with fluorescent antibodies. Isotype antibodies should be used as negative control.

### B.4 Result analysis

The results of flow cytometry analysis are analysed comprehensively with appropriate software following its user manual.

## APPENDIX C

### (Normative)

Colony‐forming unit assays

#### C.1 Instruments

C.1.1 Microscope

C.1.2 Haemocytometer

#### C.2 Reagents

C.2.1 Medium for colony‐forming unit assays: Iscove’s Modified Dulbecco’s Medium (IMDM), methylcellulose, bovine serum albumin, foetal bovine serum, β‐mercaptoethanol, L‐glutamine, supplemented with stem cell factor (SCF), granulocyte‐macrophage colony‐stimulating factor (GM‐CSF), interleukin‐3 (IL‐3), erythropoietin (EPO) and other cytokines. Store the medium at −20°C and avoid light. The medium shall be thawed overnight at 4°C before use and shall be used within 1 week.

C.2.2 IMDM: Store at 4°C and avoid light.

C.2.3 Acetic acid with methylene blue: 3%.

C.2.4 Trypan blue solution: 0.4%.

#### C.3 Procedure

C.3.1 Nucleated cell counting

Collect the cells and resuspend the cells in IMDM. Dilute the cell suspension in 3% acetic acid with methylene blue at 1:50, and mix well. Draw up 10 μl of the diluted sample and examine the density of the nucleated cells according to the method in Appendix A. Determine the concentration of the nucleated cells in suspension using the following calculations: the nucleated cells per ml = average count per square × 50 (dilution factor) × 10^4^.

C.3.2 Viable cell counting

Draw up 10 μl of the cell suspension, and determine the concentration of the viable cells in suspension using the haemocytometer (C.1.2) and microscope (C.1.1), according to the method in Appendix A.

C.3.3 Cell plating

Adjust the concentration of cell suspension to 1 × 10^4^ cells per ml. Drop up 0.5 ml of the cell suspension and dilute cells to 5 ml of the medium for colony‐forming unit assays. Vortex the tube to mix the contents thoroughly. Let stand for several minutes to allow the bubbles to rise to the top. Draw up the media containing cells into the syringe with blunt‐end needle, and dispense a volume of 1.1 ml into each 35‐mm dish. Rotate the dish to allow the medium to attach to the wall of the dish on all sides.

C.3.4 Colony formation

Incubate at 37°C, in 5% CO_2_ with ≥95% humidity for 14–16 days.

#### C.4 Analysis of results

Count and evaluate the colonies including BFU‐E, CFU‐G/M/GM and CFU‐GEMM using the microscope and the gridded scoring dish.

## APPENDIX D

### (Normative)

In vivo haematopoietic reconstruction transplantation experiment

#### D.1 Instruments

D.1.1 Microscope

D.1.2 Centrifuge

D.1.3 Flow cytometer

D.1.4 Haemocytometer

#### D.2 Reagents

D.2.1 Phosphate‐buffered saline (PBS): pH 7.4.

D.2.2 Disodium ethylenediaminetetraacetic acid solution: 1%

D.2.3 Red blood cell lysis buffer.

D.2.4 Antibodies and isotype controls. For antibody storage conditions, please refer to product instructions.

#### D.3 Procedure

D.3.1 Sample preparation

Harvest single cells by centrifugation (D.1.2) at 300 g for 5 min. Discard the supernatant. Collect the cells and resuspend the cells. Determine the concentration of the viable cells in suspension, according to the method in Appendix A. Adjust the concentration of cell suspension to 5 × 10^6^ cells per ml.

D.3.2 Cell transplantation

5.0 × 10^5^ cells were injected into sublethally irradiated (200‐300 cGy) NOD‐SCID Il2rg^null^ mice at 6 to 8 weeks of age via the tail vein, setting up a blank control group that was injected with an equal volume of phosphate buffer to the cell suspension. The number of mice per group was ≥8.

D.3.3 Peripheral blood cell collection and antibody labelling

After 16 weeks of cell injection, collect the anticoagulated peripheral blood of transplanted mice, lyse the red blood cells, and label the human CD45 antibody, CD19 antibody, CD3 antibody, CD33 antibody, CD235a antibody or the corresponding isotype control, according to the method in Appendix A. After cell incubation and washing, perform flow cytometry (D.1.3) to analyse the percentage of human CD45^+^ cells, human CD45^+^ CD19^+^ cells, human CD45^+^ CD3^+^ cells, human CD45^+^ CD33^+^ cells and human CD45^−^CD235a^+^ cells.

D.3.4 Result analysis

Using flow cytometry software to analyse the results of flow cytometry, the percentage of human CD45^+^ cells in the PBMCs is not less than 5%.

Unlabelled PBSCs from transplanted mice were used as negative control 1, and antibody‐labelled PBMCs from transplanted mice were used as biological control 2.

Firstly, according to the FSC and SSC, the target cell group 1 was gated, and the dead cells, platelets, red blood cells and cell fragments were excluded. According to the fluorescence intensity of negative control 1, the positive gate was delimited, and the positive cell group 2 was gated based on group 1.

Following the above gate setting principles, human CD45^+^ CD19^+^ cells, human CD45^+^ CD3^+^ cells, human CD45^+^ CD33^+^ cells and human CD45^−^ CD235a^+^ cells were detected. The tested positive rate minus the positive rate of control 2 is the actual positive rate of the tube. If the value is greater than 1%, it is considered positive.

